# Supplementary material for: Antimicrobial Resistance in the Global Health Network: Known Unknowns and Challenges for Efficient Responses in the 21st Century
Source: Microorganisms. 2023 Apr 17;11(4):1050. doi: 10.3390/microorganisms11041050 (PMC10144039; doi:10.3390/microorganisms11041050)
Supplement: Supplementary file 1 [file microorganisms-11-01050-s001.zip › microorganisms-1941860-supplementary.pdf]

Supplementary Materials

Table S1. AMR definitions, applications, and gaps [220].

AMR definition type	Definition	Application	Comments
Clinical [221]	A microorganism is categorized as "resistant" when there is a high likelihood of therapeutic failure even when the highest approved dose is used.	Human/veterinary bacteria and antimicrobials used for human or animal therapy	Not valuable for detecting resistance mechanisms for treating real patients or animals, for "non-human bacteria," or for which antibiotic breakpoint concentrations have not been established.
Epidemiological [222]	Isolates in which the corresponding MIC value for an antimicrobial is higher than that of the upper limit of a particular species' wild-type ("normal") population. Subpopulations with higher MICs are suspicious of harboring "acquired" resistance mechanisms. Such MIC values are known as "*epidemiologic cutoff values* ECOFF (or ECV by the CLSI).	Any antimicrobial including non-antibiotic antimicrobials as biocides.	It can be applied to clinical and environmental isolates once MIC distributions have been established over independent isolates. Enables the detection of either low-level or high-level "acquired" resistance mechanisms
Operational [220]	The differences in susceptibility after pairwise comparison of a parental strain with either an isogenic mutant strain or a strain containing an acquired ARG by HGT in laboratory conditions.	Functional genomics and functional metagenomics for clinical (human and veterinary) or environmental strains	Useful for deciphering AMR mechanisms
Ecological [220,223]	The function that ARGs exert in the environment. It implies genes that evolved in nature to counteract their production of antimicrobials or production by neighboring bacteria and those acquired by HGT after introducing antibiotics in therapeutics.	Ecological and evolutionary (eco-evo) studies<br>Used for metagenomics (resistome) [63]	It can be applied to both ARGs present in antibiotic-producing microorganisms (e.g. *Streptomyces* and aminoglycoside resistance genes) or ARGs present in non-antibiotic-producing microorganisms (e.g., *Kluyvera* spp. and *bla*$_{CTX-M}$)

Abbreviations. MIC, minimum inhibitory concentration; CLSI, Clinical and Laboratory Standards Institute; AMR= antimicrobial resistance; ARG, antibiotic-resistant gene; HGT, horizontal gene transfer. (*) which often constitutes a gateway for acquiring high-level clinically relevant resistance.

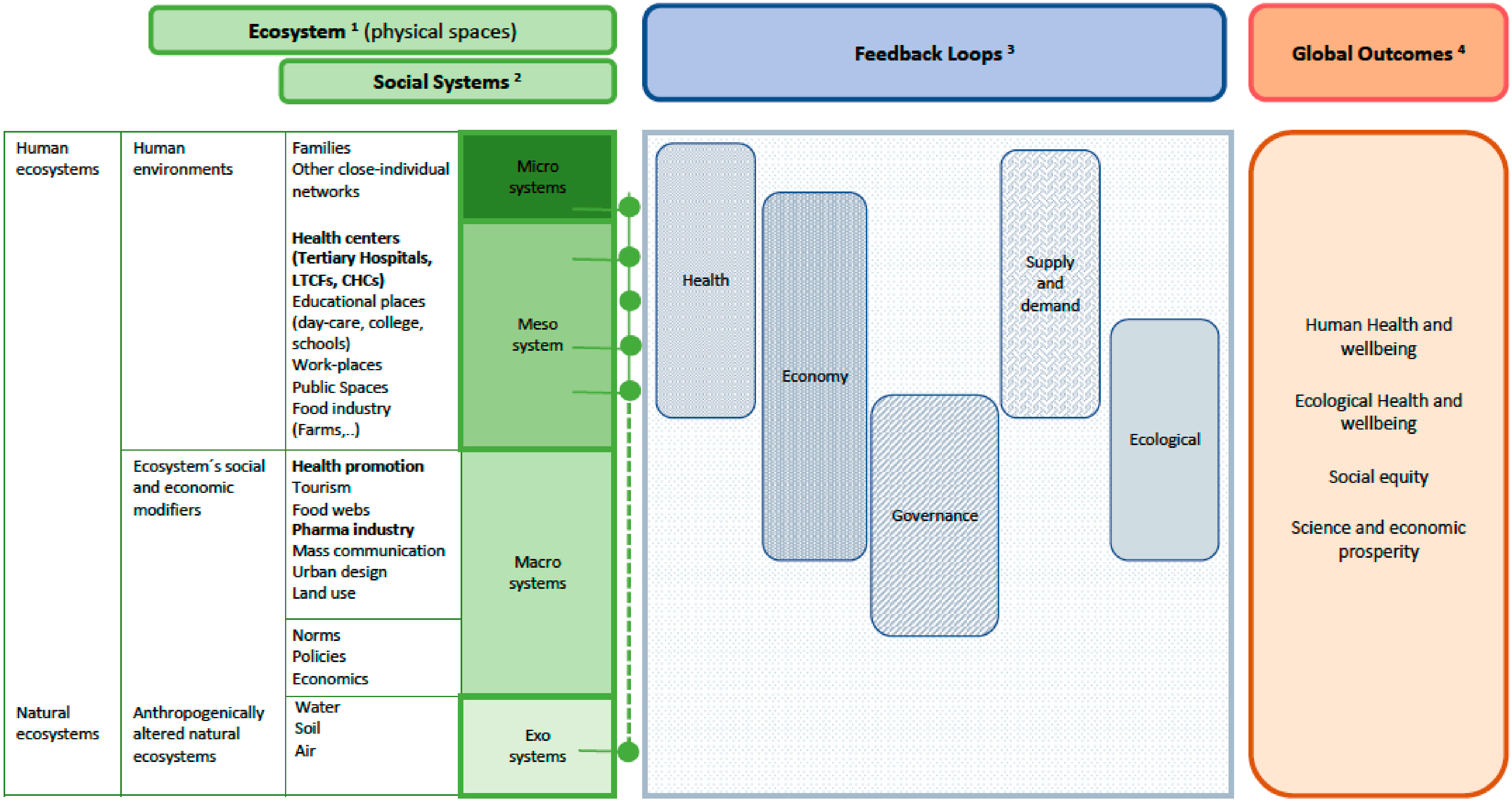

Figure S1. Antimicrobial resistance in the healthcare sector from a systems perspective. Adapted from [91]. Also influenced by "The Convergence Model," which established major interlocking domains of the determinants of the emergence of infection: genetic and biological factors; physical environmental factors; ecological factors; and social, political, and economic factors [33], Lewontin ideas [40] and the "Bronfenbrenner´s bioecological system model" of human development [113,114]. The "systems perspective" (a metasystem of nested systems) is adopted to understand and address the underlying drivers of various global public health issues that co-exist with others. In this scenario, people influence the various system loops at individual and group levels. Such an influence is always bidirectional (people influence the system/level and the system/level influences people) and synergistic, with an impact that depends on the degree of interactions. We focus on the antimicrobial resistance problem in the multilayered health sector, which is part of a significant social and physical metasystem.

[1] **Ecosystems**. They overlap with "social systems" in the bioecological model (see below). [2] **Social systems (factors)** determine community health. The "*Bronfenbrenner's bioecological system model"* is an evolving theoretical system for the scientific study of human development (behavior) over time. It relies on four components (persons, processes, context, and time) and their relationships (interactions and connections). The model establishes different layers (micro-, meso-, exo-, macro-, and chronosystems) for ordering these components and relationships. *Microsystem*: the immediate environment (e.g., family); *mesosystem*: influence from the relationships with microsystems; *exosystem*, the relationship between microsystems and other systems with sporadic exposure; *macrosystems*, larger cultural and social contexts. The chronosystem refers to the time when events occur and the larger historic landscape. Changes or conflicts in any one layer will ripple throughout other layers. This theory has recently been renamed "bioecological systems theory" to emphasize that the own biology (age, sex) is a primary environment fueling human development in agreement with some evolutionary frames [40]. These layers parallel those of microbial fluxes [3,4,31,38,61,165]. In the context of AMR and infectious diseases, social factors are relevant for approaching antibiotic consumption, adopting WASH measures, or sanitation [12,13,104]. It focuses on people as central to development and concerns all surrounding factors to progress and achieve a better life for a person and a community as its ultimate goal. [3] **Feedback loops** are governance feedback loops that determine how political power translates into policies, economic incentives, and disincentives for improving healthcare institutions. *Health business and economy feedback loops* determine the dynamics for creating profitable goods and services, including the externalities associated with damage to human health, the environment, and the planet; *Supply and demand feedback loops* show the relationships that determine current antibiotic consumption practices; *Ecological feedback loops* show the unsustainable effects that antibiotic use and resistance impose on health and natural ecosystems; and *Human health feedback loops* show the positive and negative impacts that these effects have on human health systems. *Governance Loops*. According to cultural and personality dimension models, Western countries with low and high antibiotic consumption show distinct and marked national culture patterns. However, *governance quality* is essential to proper antibiotic use [12]. A more detailed analysis of truly "collectivistic" countries (individuals living integrated into primary groups) and "long-term oriented" societies (more pragmatic, tolerant, and oriented to future rewards) are necessary for a better understanding of the human response to ailments, especially epidemics, and antibiotic use [33,40,32]. [4] Four primary ***broad global outcomes***, human and animal health and well-being (OneHealth), ecological Health and well-being (Planetary Health), and Social equity and economic prosperity (Global Health), are all related to AMR [113,107].
